# Case report and literature analysis: pancreatic hepatoid carcinoma with multiple lymph node metastases progressing to liver metastasis after pancreaticoduodenectomy

**DOI:** 10.3389/fonc.2024.1335647

**Published:** 2024-04-26

**Authors:** Xiaorui Huang, Xinyi Guo, Yahong Yu

**Affiliations:** ^1^ Department of Biliopancreatic Surgery, Tongji Hospital, Tongji Medical College, Huazhong University of Science and Technology, Wuhan, China; ^2^ Hubei Key Laboratory of Hepato-Biliary-Pancreatic Diseases, Tongji Hospital, Tongji Medical College, Huazhong University of Science and Technology, Wuhan, China

**Keywords:** pancreatic hepatoid carcinoma, postoperative liver metastases, immunotherapy, case report, literature analysis

## Abstract

Hepatoid carcinoma is an extrahepatic primary tumor displaying characteristics reminiscent of hepatocellular carcinoma differentiation, which is found in various organs, such as the stomach, ovaries, gallbladder, and pancreas. Reports of pancreatic hepatoid carcinoma remain scarce. Consequently, understanding of this disease remains a priority, with no established consensus on its diagnosis and management. Here, we reported the case of a 45-year-old woman diagnosed with hepatoid carcinoma located in the pancreatic head, accompanied by multiple lymph node metastases. Following pancreaticoduodenectomy, the patient developed liver metastases within 3 months. Subsequently, she underwent adjuvant therapy consisting of Teysuno and Durvalumab following microwave ablation for the liver metastases. Remarkably, the patient has survived for one year without significant disease progression. This case underscores the potential efficacy of immunotherapy as a promising treatment option for pancreatic hepatoid carcinoma. Further research and clinical trials are warranted to explore the optimal management strategies for this rare and challenging malignancy.

## Introduction

Pancreatic hepatoid carcinoma (HC) represents a distinct carcinoma variant exhibiting hepatocellular carcinoma (HCC)-like features, often associated with elevated serum alpha-fetoprotein (AFP), and remains uncommon in clinical practice (1). Prone to vascular invasion and liver or lymph node metastasis, HC is considered a highly aggressive malignant tumor with a poor prognosis (2). Despite being first described in 1987 (3), only around 41 cases have been documented in the literature to date (4). However, comprehensive large-scale clinical investigations on pancreatic HC are still notably lacking, leading to a lack of consensus regarding its diagnosis and treatment protocols.

In this study, we present a case of pancreatic HC accompanied by multiple lymph node metastases, followed by early liver metastases post-pancreaticoduodenectomy. The patient underwent adjuvant therapy with Teysuno (also known as S-1) alongside immunotherapy utilizing Durvalumab. This case underscores the urgent need for further research and clinical exploration to establish standardized diagnostic and therapeutic approaches for pancreatic HC.

## Case description: diagnosis and treatment

**Figure 1:**
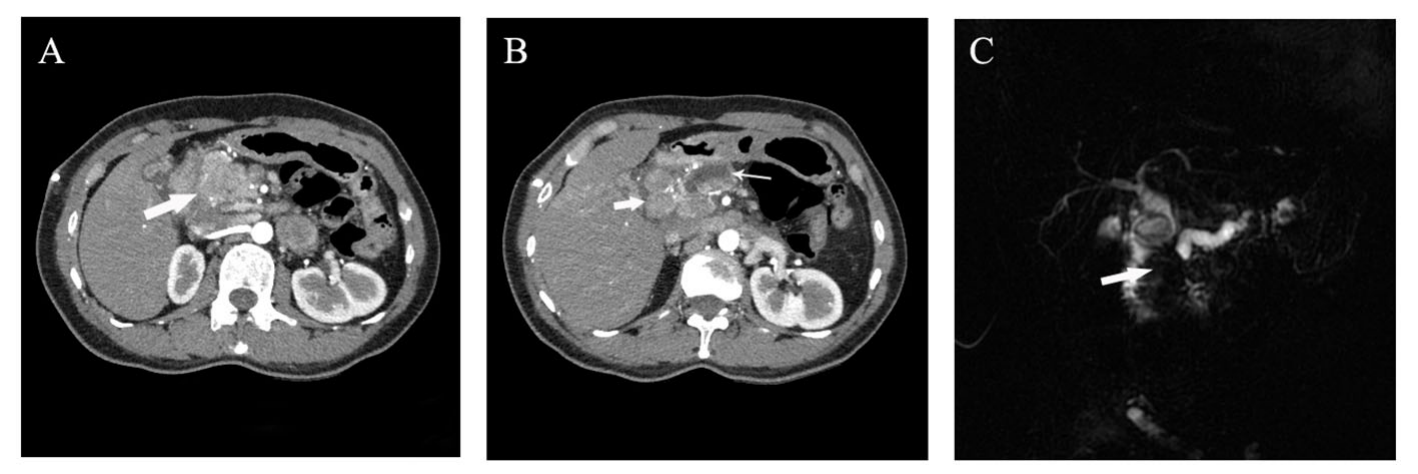
Preoperative imaging examination of the patient. **(A, B)** Abdominal arterial phase CT imaging suggested the presence of a 4 cm irregular mass with inhomogeneous enhancement in the pancreatic head (denoted by thick arrow in **A**), along with enlarged lymph nodes around the head of the pancreas (approximately 2cm in diameter, thick arrow in **B**) and an dilated main pancreatic duct with maximum width of 8mm (denoted by thin arrow in **B**); **(C)** Magnetic resonance imaging indicated truncation of both the common bile duct and the main pancreatic duct resulting from a mass in the pancreatic head.

A 45-year-old Chinese woman was admitted to our center in 2022 for abdominal pain accompanied by jaundice. The patient has a history of chronic Viral hepatitis B without receiving regular antiviral therapy. The results of laboratory tests after admission were as follows: the quantification of hepatitis B virus DNA: 4.25×102 IU/mL, alpha fetoprotein (AFP): 5229.00 ng/mL, protein induced by vitamin K absence or Antagonist-II (PIVKA-II): 176.32 mAu/mL, carbohydrate antigen 19-9 (CA19-9): 12.95 U/mL, carcinoembryonic antigen (CEA): 1.00 ng/mL. Abdominal enhanced computed tomography (CT) and magnetic resonance imaging (MRI) revealed an irregular mass measuring approximately 47×35mm in the head of the pancreas, with uneven abnormal enhancement characteristics. Additionally, multiple lymph nodes in the hilar and retroperitoneal regions were found to be enlarged, along with dilation of the distal pancreatic duct, upper common bile duct, and intrahepatic bile ducts (**Figure 1**). Consequently, percutaneous transhepatic cholangial drainage (PTCD) was performed on the patient to alleviate jaundice. Furthermore, endoscopic ultrasound (EUS) and fine needle aspiration biopsy (FNA) were carried out. The subsequent pathological examination suggested the presence of a pancreatic malignant tumor, possibly a poorly differentiated AFP-producing carcinoma, high-grade neuroendocrine tumor, or pancreatic acinic cell carcinoma.

**Figure 2:**
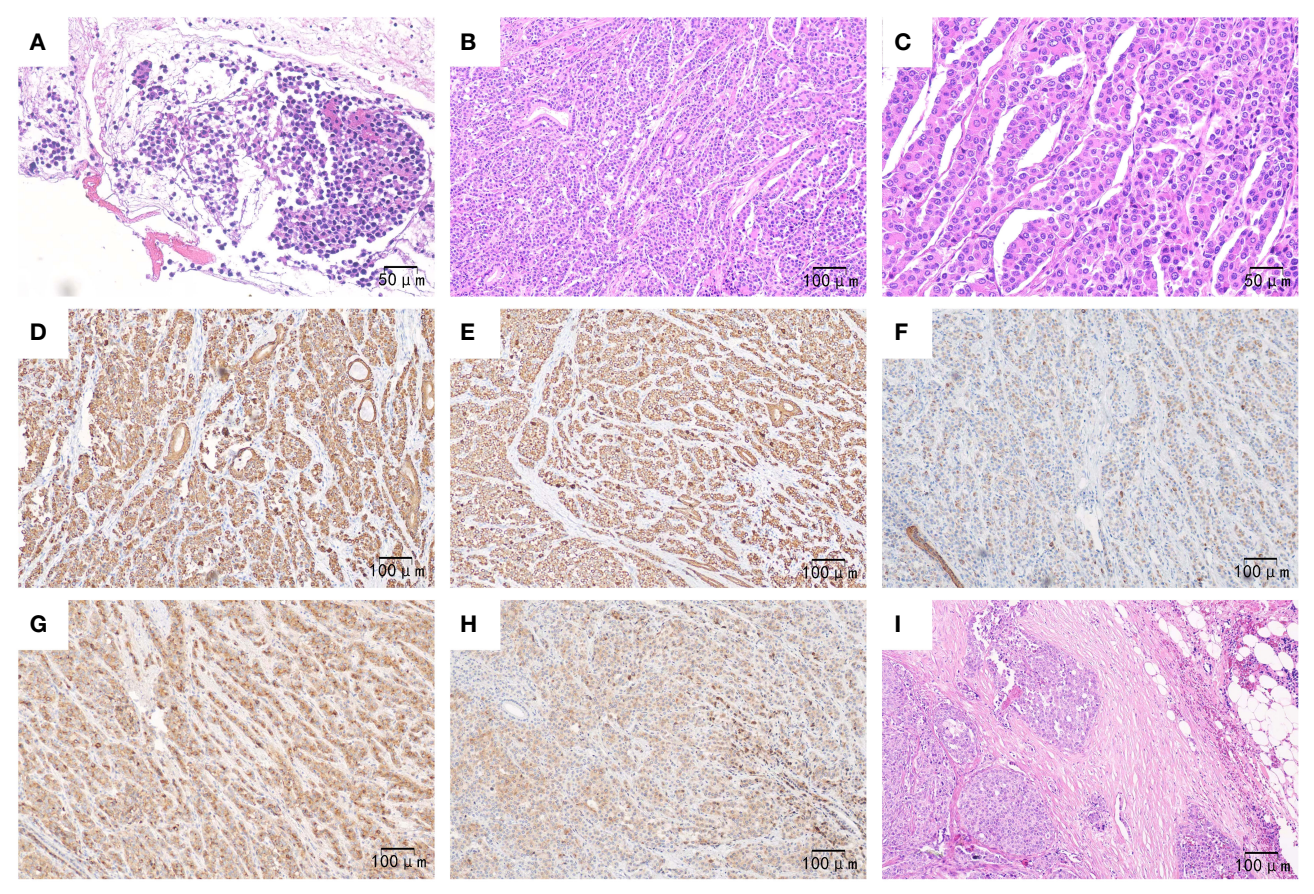
Pathological outcomes of the patient. Preoperatively, **(A)** (×200): Hematoxylin-eosin staining revealed heteromorphic tumor cells in the pancreas tissue obtained from fine needle aspiration. Postoperatively, **(B)** (×100): Hematoxylin-eosin staining showed that the poorly differentiated tumor exhibited hepatoid differentiation, with neoplastic cells growing in a sheet-like or trabecular pattern along with sinusoids. **(C)** (×200): Hematoxylin-eosin staining demonstrated polygonal or cubic eosinophilic cells with abundant cytoplasm and enlarged, prominent nuclei. **(D–H)** (×100): Immunohistochemistry staining revealed tumor cells expressing CK7, CK8/18, CK19 (with weak positivity), AFP, and Glypican-3, respectively; **(I)** (×100). Hematoxylin-eosin staining displayed heteromorphic tumor cells proliferating within the lymph node.

Eventually, the patient underwent pancreaticoduodenectomy and abdominal lymph node dissection after the jaundice had subsided. The final pathological examination confirmed the presence of poorly differentiated pancreatic hepatoid adenocarcinoma invading the mucosal layer of the duodenal wall. Additionally, there were multiple peripancreatic lymphatic node metastases (12/12). The immunohistochemical results were as follows (**Figure 2**): AFP (+), Glypican-3 (+), Hep Par-1(-), Arginase-1 (-), CK7 (+), CK8/18 (+), CK19 (+), CK20 (-), E-Cadherin (+), EMA(+), Vimentin (-), β-catenin (+), P53 (+), RB1 (+), Ki-67 (Li approximately 40%). Thereby, the patient was diagnosed with advanced pancreatic HC, staged at T4N2M0, following the 8th AJCC/UICC staging system for pancreatic ductal adenocarcinoma.

The patient recovered well from the surgery, and her serum AFP levels returned to normal. And then, the patients started combination chemotherapy with gemcitabine and albumin-bound paclitaxel a month after surgery. However, after undergoing standard chemotherapy for about 2 months, the patient exhibited elevated serum AFP levels (591.80 ng/mL) and the presence of scattered round nodules in the liver on the MRI which were considered as metastatic lesions.

Sequentially, ultrasound-guided microwave ablation was performed to treat the newly discovered liver masses. Considering the patient’s weak physical condition and intolerance to prior chemotherapy regimen, Teysuno monotherapy was recommended to replace previous adjuvant chemotherapy (gemcitabine plus albumin-bound paclitaxel). To better eradicate any potential residual metastatic lesions, durvalumab was added as an exploratory treatment. Throughout the treatment process, the patient complained of no serious discomfort, although the laboratory test suggested mild hypothyroidism. Consequently, levothyroxine supplementation and regular thyroid function testing were provided. Positron emission tomography/computed tomography (PET/CT) the patient received five months after chemotherapy plus immunotherapy indicated that there was no significant increase in liver and retroperitoneal lymph nodes’ metabolic activity, suggesting that the tumor activity was basically suppressed. Additionally, the patient’s serum AFP level has decreased to 90.66ng/mL. It is worth mentioning that the patient has successfully survived for one year after the surgery.

## Discussion

Since its first report by Ishikura et al. in 1970 (5), scholars have subsequently discovered that hepatoid carcinoma can develop in various organs besides the stomach, including the esophagus, lung, gallbladder, pancreas, colorectum, ovary, and others (1). However, reports of pancreatic hepatoid carcinoma remain relatively rare even in present times. Consequently, there is still a lack of relevant studies on the unique pathogenesis and progression mechanisms of pancreatic HC. Additionally, there is no international consensus on the standardized diagnosis and treatment of this disease. Therefore, further exploration is needed to enhance our clinical understanding of pancreatic HC.

Excluding cases diagnosed with pancreatic ectopic hepatocellular carcinoma (HCC) and AFP-producing pancreatic carcinoma, a total of 42 cases of pancreatic HC were reported in the literature. These cases have been pooled and summarized in **Tables 1**, **2**. The majority of these patients were male (69.05%) and older than 50 years (57.14%).Their ages ranged from 21 to 83 years, with a median age of 53 years. The most common clinical manifestation among these patients was abdominal and/or back pain (30.95%), followed by weight loss (19.05%) and jaundice (16.67%). Approximately one-third of these patients were asymptomatic. 19 out of the 31 patients (61.29%) who underwent preoperative serum AFP examinations were found to have elevated levels. On the other hand, only 24.13% of the patients (7/29) with available preoperative data on serum CEA showed elevated levels. Among these cases, the majority (54.76%) of the tumors were located in the body or tail of the pancreas, and 40.48% were found to have other histological components alongside, including neuroendocrine tumors (10/42), ductal adenocarcinoma (3/42), serous cystic adenoma (2/42), and acinar cell carcinoma (1/42). Additionally, 42.86% (18/42) of the patients had metastatic lesions, with liver metastases observed in 13 cases.

**Table 1:** Summary of clinical characteristics of 42 cases of pancreatic hepatoid carcinomas reported in the literature.

No.	Report	Age	Sex	Clinical presentation	AFP level	CEA level	Location	Size(cm)	Concurrent component	Metastasis	Treatment	Follow-up (month)	Survival
1	Hruban et al, 1987 (3)	53	F	Subcutaneous fat Necrosis and polyarthritis	Normal	NA	Tail	1	Acinar cell carcinoma	Yes	Chemotherapy (5-FU, Adriamycin)	2.75	Died
2	Yano et al, 1999 (6)	57	M	Jaundice, epigastric pain vomiting and fever	Elevated	Elevated	Head	9×7×5	PDAC	No	Surgery	3	Died
3	Tanno et al, 1999 (7)	65	F	Epigastric and back pain, anorexia and weight loss	Elevated	Elevated	Body-tail	6×5	PDAC	Yes	Palliative care	6	Died
4	Paner et al, 2000 (8)	28	M	Abdominal and back pain	NA	NA	Multifocal	8×8×6	PDAC	Yes	Surgery, chemotherapy	14	Died
5	Paner et al, 2000	57	M	Vomiting diarrhea, weight loss,diffuse skin rashes and diabetes mellitus	NA	NA	Tail	6×4×3.5	NET	Yes	Surgery, chemotherapy	102	Died
6	Lam et al, 2001 (9)	64	F	Hypoglycemia, Recurrent nocturnal sweating	Elevated	NA	Tail	7×4×4	NET	Yes	Surgery, regional Embolization, systemic chemotherapy	22	Died
7	Cuilliere et al, 2002 (10)	70	M	Asymptomatic	Normal	Normal	Body	3	SCN	No	Surgery	12	Alive
8	Hughes et al, 2004 (11)	51	M	Asymptomatic	Normal	NA	Tail	6×5.5×5.5	No	No	Surgery	14	Alive
9	Matsueda et al, 2006 (12)	49	F	Weight loss	Elevated	Normal	Widespread	NA	No	Yes (Postoperatively)	Surgery, chemotherapy (gemcitabine)	48	Alive
10	Shih et al, 2006 (13)	32	M	Asymptomatic	Normal	Normal	Tail	7	No	No	Surgery	18	Alive
11	Oh et al, 2006 (14)	21	M	Asymptomatic	Elevated	NA	Head	3.3×2.5×2.5	NET	No	Surgery	7	Alive
12	Hameed et al, 2007 (15)	41	F	Gastroesophageal reflux, jaundice, abdominal pain	Elevated	NA	Head	4.5×4×3	NET	Yes	Surgery, Chemotherapy (cisplatin and irinotecan)	27	Died
13	Liu et al, 2007 (16)	80	M	Nausea, diarrhea weight loss and epigastric palpable mass	Normal	Normal	Head	5×5×6	No	Yes	Surgery	8	Alive
14	Zhang et al, 2007 (17)	37	F	Upper abdominal pain, anorexia, and emaciation	Elevated	Elevated	Widespread	9	NET	No	Surgery	3	Died
15	Jung et al, 2010 (18)	46	M	Dyspepsia, epigastric palpable mass	Elevated	Normal	Head	8×9	NET	No	Surgery	4	Alive
16	Petrelli et al, 2012 (19)	37	F	Epigastric abdominal mass	NA	Normal	Body	11	No	Yes	Targeted therapy (sorafenib)*	12	Died
17	Kelly et al, 2012 (20)	53	F	Epigastric pain	Elevated	NA	Body-tail	NA	No	Yes	Surgery, Chemotherapy (carboplatin and gemcitabine)	22	Alive
18	Kai et al, 2012 (21)	79	F	Asymptomatic	**NA**	**Normal**	Tail	7×5	No	Yes	Surgery	2	Died
19	Huang et al, 2012 (22)	52	M	Jaundice, anorexia, and epigastric pain	NA	Elevated	Head	0.5 nodule	NET	No	Surgery, chemotherapy(sunitinib)	16	Alive
20	Majumder et al, 2013 (23)	60	M	Left upper quadrant pain, jaundice and nausea	Normal	NA	Head	5.8×6.0	No	Yes	Biliary drainage, chemotherapy(GEM)	3	Died
21	Xin et al, 2014 (24)	33	F	Asymptomatic	Elevated	Normal	Head	2×1.4×1.8	NET	No	Surgery, chemotherapy(GEM)	46	Alive
22	Vanoli et al, 2015 (25)	57	F	Jaundice	Elevated	Elevated	Head	3.5×3×3	Lymphoid stroma	No	Surgery, chemotherapy(GEM)	10	Alive
23	Soofi et al, 2015 (26)	69	M	Atypical chest pain	Elevated	Normal	Body-Tail	5.9	No	No	Surgery	4	Alive
24	Antonini et al, 2015 (27)	59	M	Weight loss, abdominal discomfort	Normal	Normal	Body	6×5	No	No	Targeted therapy (sorafenib)	4	Died
25	Kuo et al, 2015 (28)	67	M	Asymptomatic	Normal	Normal	Tail	2×2	No	No	Surgery	6	Alive
26	Veeran- kutty et al, 2015 (29)	47	M	Asymptomatic	NA	Normal	Tail	3.1×2.9×2.6	SCN	No	Surgery	8	Alive
27	Williams et al, 2015 (30)	71	M	Pancreatitis, melena (oozing ulcer at the ampulla)	Normal	Normal	Head	5	No	Yes	Surgery	NA	NA
28	Stamatova et al, 2016 (31)	78	M	Asymptomatic	Normal	Normal	Head	8×6	No	No	Surgery	2	Died
29	Chang et al, 2016 (32)	61	M	Asymptomatic	NA	NA	Body-Tail	1.3	No	No	Surgery	6	Alive
30	Akimoto et al, 2016 (33)	59	M	Asymptomatic	Normal	Normal	Body	5.0×3.5	No	No	Surgery	12	Alive
31	Pellini Ferreira et al, 2017 (34)	43	M	Jaundice, epigastric pain, and watery diarrhea	Normal	Normal	Tail	9.0	NET	Yes	Chemotherapy (capecitabine and temozolomide)	16	Alive
32	Ma et al, 2017 (35)	75	M	Weight loss	Elevated	Elevated	Tail	7.8	No	Yes	Neoadjuvant chemotherapy, Surgery	10	Alive
33	Fukushia et al, 2018 (36)	44	M	Asymptomatic	NA	Normal	Head	1.9×1.8	No	No	Surgery	NA	NA
34	Yang et al, 2018 (2)	83	M	Abdominal pain	NA	NA	Body	2.7×2.5×1.5	No	No	Surgery	105	Alive
35	Yang et al, 2018 (2)	72	M	Severe back pain	NA	NA	Tail	12×10.5×4.5	No	No	Surgery	1	Died
36	Yang et al, 2018 (2)	54	M	Asymptomatic	Elevated	Normal	Body-Tail	10×9×9	No	No	Surgery	29	Died
37	Tomino et al, 2019 (37)	56	M	Asymptomatic	NA	Normal	Head	0.7	No	No	Surgery	6	Alive
38	Zeng et al, 2020 (38)	36	M	Painless jaundice and weight loss	Elevated	Normal	Widespread	6.0×7.0	No	No	Palliative care	4	Died
39	He et al, 2021 (39)	44	F	Upper abdominal pain, vomiting	Elevated	Normal	Body-Tail	7.6×7.3	No	No	Surgery, TACE, Immunotherapy (Carrilizumab)	8	Alive
40	Trinh et al, 2021 (40)	49	M	abdominal pain	Elevated	Normal	Head	2.6×2.8	NET	Yes	Surgery, chemotherapy	12	Alive
41	Wei et al, 2023 (4)	48	M	Asymptomatic	Elevated	NA	Head	7.5×7×3.7	No	Yes	Surgery, Targeted therapy(Sorafenib)	8	Alive
42	Our case	45	F	Upper abdominal Pain, jaundice	Elevated	Elevated	Head	4.7×3.5	No	Yes	Surgery, chemotherapy	12	Alive

M, male; F, female; PDAC, pancreatic ductal adenocarcinoma; NET, neuroendocrine tumors; SCN, serous cystic neoplasia; NA, not available.

**Table 2:** Clinical and biochemical characteristics of the 42 cases of pancreatic HC.

Variable	Number of cases (%)
Age Median(range)	53(21-83)yr
≥ 50yr	24(57.14)
<50yr	18(42.86)
Sex	
Male	29(69.05)
Female	13(30.95)
Clinical presentation	
Asymptomatic	15(35.71)
Abdominal/back pain	13(30.95)
Weight loss	8(19.05)
Jaundice	7(16.67)
Abdominal mass	3(7.14)
Location	
Head	15(35.71)
Body or tail	23(54.76)
Widespread	4(9.52)
Serum AFP level	
Elevated	19(45.23)
Normal	12(28.57)
NA	11(26.19)
Serum CEA level	
Elevated	7(16.67)
Normal	22(52.38)
NA	13(30.95)
Concurrent component	
No	25(59.52)
Neuroendocrine tumors	10(23.81)
Ductal adenocarcinoma	3(7.14)
Acinar cell carcinoma	1(2.38)
Other	3(7.14)
Metastasis	18(42.86)
Liver	13(30.95)
Lymph node	4(9.52)
Treatment	
Surgery only	21(50.0)
Surgery combining other adjuvant therapy	14(33.33)
Others	7(16.67)
Outcome	
Alive	24(57.14)
Death	16(38.10)
NA	2(4.76)

HC, Hepatoid carcinoma, AFP, Alpha-fetoprotein; CEA, Carcinoembryonic antigen;

NA, not available.

In a retrospective study of 271 patients with HC in various organs, it was found that 84.8% of patients had elevated serum AFP levels (41). Therefore, initially HC was thought to be an AFP-producing tumor (42). However, it is important to note that not all cases of HC express AFP, and likewise, AFP-producing tumors are not always linked to hepatoid differentiation. Generally, AFP-producing tumors are defined as those with elevated serum AFP or positive expression of AFP in immunohistochemistry examination, whereas HC is a tumor that exhibits hepatocellular carcinoma differentiation features (43). Although serum AFP levels are typically elevated in most cases of pancreatic HC, it has been reported that elevated serum AFP can also be observed in other types of pancreatic tumor, such as acinar cell carcinoma (16), ductal adenocarcinoma (44), neuroendocrine tumor (45), and pancreatoblastomas (46). As demonstrated in our case, serum AFP can serve to evaluate the effectiveness of treatment and identify tumor recurrence during follow-up (47).

Lacking characteristic imaging features, the diagnosis of pancreatic HC heavily relies on histopathology. Similar to HCC, the typical hepatoid differentiation area of pancreatic HC comprises polygonal or cubic eosinophilic cells growing in a sheetlike or trabecular pattern with sinusoids with abundant cytoplasm and enlarged, prominent nuclei. The presence of bile production, which serves as substantial evidence of hepatocyte lineage differentiation, is a more definitive diagnostic feature (1). Commonly, immunohistochemistry is employed in the differential diagnosis of pancreatic HC. A series of hepatocyte markers are expressed in pancreatic HC, including AFP, Hepatocyte antigen (Hep Par1), Glypican-3, and Arginase-1, with positivity rates of 67%, 96%, 78%, and 75% respectively (37). Actually, pancreatic HC needs to be distinguished from other types of pancreatic cancer such as ductal adenocarcinoma, acinar cell carcinoma, neuroendocrine carcinoma, etc. Additionally, it must also be differentiated from pancreatic metastases of HCC or HCC originating from ectopic liver tissues, which may be more common (with an incidence of 2.7% to 5.6%) than primary pancreatic HC (48). In our case, the diagnosis of pancreatic HC could not be confirmed based on the preoperative EUS-FNA pathology, as it is challenging to differentiate it from acinar cell carcinoma or neuroendocrine carcinoma. Since no obvious intrahepatic neoplastic lesions were detected on the preoperative imaging(both enhanced CT and MRI), the diagnosis of pancreatic HC was formally established, based on the combination of microscopic morphological features and immunohistochemical results after thorough postoperative pathological examination.

83.33% (35/42) of patients reported in the available literature received surgical treatment, demonstrating the superiority of surgery over non-surgical treatment. Generally, radical surgical resection is considered the preferred treatment option (23). Although there are few relevant studies currently, adjuvant chemotherapy may be a viable treatment to improve the prognosis for those instances that are inoperable. The role of targeted therapy and immunotherapy in this disease is still uncertain. Similar pathomorphologic features suggest that the biological behavior of pancreatic HC may share more similarities with HCC. Several reports have highlighted the success of sorafenib in treating pancreatic HC, indicating that sorafenib may offer short-term therapeutic advantages for patients with unresectable metastatic pancreatic HC (19). He et al. reported that a patient with pancreatic HC received transarterial chemoembolization (TACE) combined with postoperative immunotherapy (karelizumab), which brought about positive outcomes (relief of abdominal symptoms, a gradual decrease in serum AFP, and no recurrence observed at the 8-month follow-up) (39). Durvalumab, as one of the PD-L1 inhibitors, is mainly applied to the treatment of non-small cell lung cancer and bile tract cancer. Recently, studies on the role of Durvalumab in hepatocellular carcinoma and pancreatic cancer have been ongoing (49, 50). Basso et al. reported a case of hepatoid adenocarcinoma of the lung responding to PD-L1 inhibitor (durvalumab) therapy despite no PD-L1 expression (51). After a multidisciplinary discussion, the patient was recommended to receive durvalumab immunotherapy on the basis of S1 single-agent chemotherapy following microwave ablation for liver metastases, even though pertinent testing indicated PD-L1 negative. The patient is still in close follow-up with no significant progress. Indeed, there is a lack of validated markers to accurately predict tumor responsiveness to immunotherapy. The exact role of PD-L1 inhibitor in the HC is also unclear. Regardless, it is an exploration in the treatment of HC, and more research is required to evaluate the role of immunotherapy in HC.

## Conclusion

The case we reported indicated that the combination of microwave ablation with adjuvant therapy of S1 and durvalumab postoperatively might improve the prognosis and quality of life, providing a potential option for HC.

## Data availability statement

The original contributions presented in the study are included in the article/supplementary material. Further inquiries can be directed to the corresponding author.

## Ethics statement

The studies involving humans were approved by the institutional review board of the Tongji Hospital, Tongji Medical College, Huazhong University of Science and Technology. The studies were conducted in accordance with the local legislation and institutional requirements. Written informed consent for participation was not required from the participants or the participants’ legal guardians/next of kin in accordance with the national legislation and institutional requirements. Written informed consent was obtained from the individual(s) for the publication of any potentially identifiable images or data included in this article.

## Author contributions

XH: Writing – review & editing, Writing – original draft, Methodology, Investigation. XG: Writing – review & editing, Investigation, Data curation. YY: Writing – review & editing, Supervision, Resources, Funding acquisition.
